# Supplementary material for: tRNA epitranscriptomics and biased codon are linked to proteome expression in Plasmodium falciparum
Source: Mol Syst Biol. 2018 Oct 4;14(10):e8009. doi: 10.15252/msb.20178009 (PMC6171970; doi:10.15252/msb.20178009)
Supplement: Supplementary file 1 — Appendix [file MSB-14-e8009-s001.docx]

**Appendix**

**tRNA epitranscriptomics and biased codon usage are linked to proteome expression in *Plasmodium falciparum***

Chee Sheng Ng, Ameya Sinha, Yaw Aniweh, Qianhui Nah, Indrakanti Ramesh Babu, Chen Gu, Yok Hian Chionh, Peter C. Dedon, Peter R. Preiser

**Table of Contents**

• **Figure S1**: Step-wise extraction, isolation, and quality examination of *P. falciparum* tRNA. *Related to Figures 1 and 2.*

• **Figure S2**: Proteomic analysis of the *P. falciparum* IDC time-course. *Related to Figure 3*.

• **Figure S3**: *Plasmodium falciparum* Gly-tRNA^GGA^ contains wobble mcm^5^U. *Related to Figure 6.*

• **Figure S4**: Relative abundance of glutamic acid (GAA), glycine (GGA), and glutamine (CAA)

isoacceptors as quantified by LCMS across ring, trophozoite and schizont stage of the parasite’s asexual lifecycle. *Related to Figure 5.*

• **Table S1**: Modified ribonucleosides identified by LC-MS/MS analysis of *P. falciparum* tRNA hydrolysates. *Related to Figure 1.*

• **Table S2**: Genome average usage for codons enriched in up- and down-regulated at 46 hr of the IDC. *Related to Figure 3.*

• **Table S3**: Enriched synonymous codons and their under-represented counterparts in late-stage (TP 40 and 46) up-regulated proteins. *Related to Figure 2 and 4.*

• **Table S4**: Electrospray series for unmodified and modified tRNA species presenting summary data for the T1-digested *Plasmodium falciparum* tRNA. *Related to Figure 6.*

**• Table S5.** Codon usage and cytoplasmic tRNAs in *P. falciparum*.

**• Table S6(a).** Normalized Signal Intensities for tRNA modifications measured over six time points*.* **(b)** tRNA modification fold-change values calculated from normalized mass spectrometer signals relative to an arbitrary average over the six time points. **(c)** Statistical analysis for tRNA modification analysis. **(d)** Signal Intensities for mapped tRNA modifications measured over three time points. **(e)** tRNA Normalized Signal Intensities for mapped tRNA modifications measured over three time points. Table included as an additional file in Ng et al Appendix Table S6.xsxl

**• Table S7.** Proteomics data. Table included as an additional file in Ng et al Appendix Tables S7.xsxl

**• Table S8.** Translation efficiency calculations. Table included as an additional file in Ng et al Appendix Table S8.xsxl.

**• Table S9.** Genome-wide codon usage data. Table included as an additional file in Ng et al Appendix Table S9.xsxl


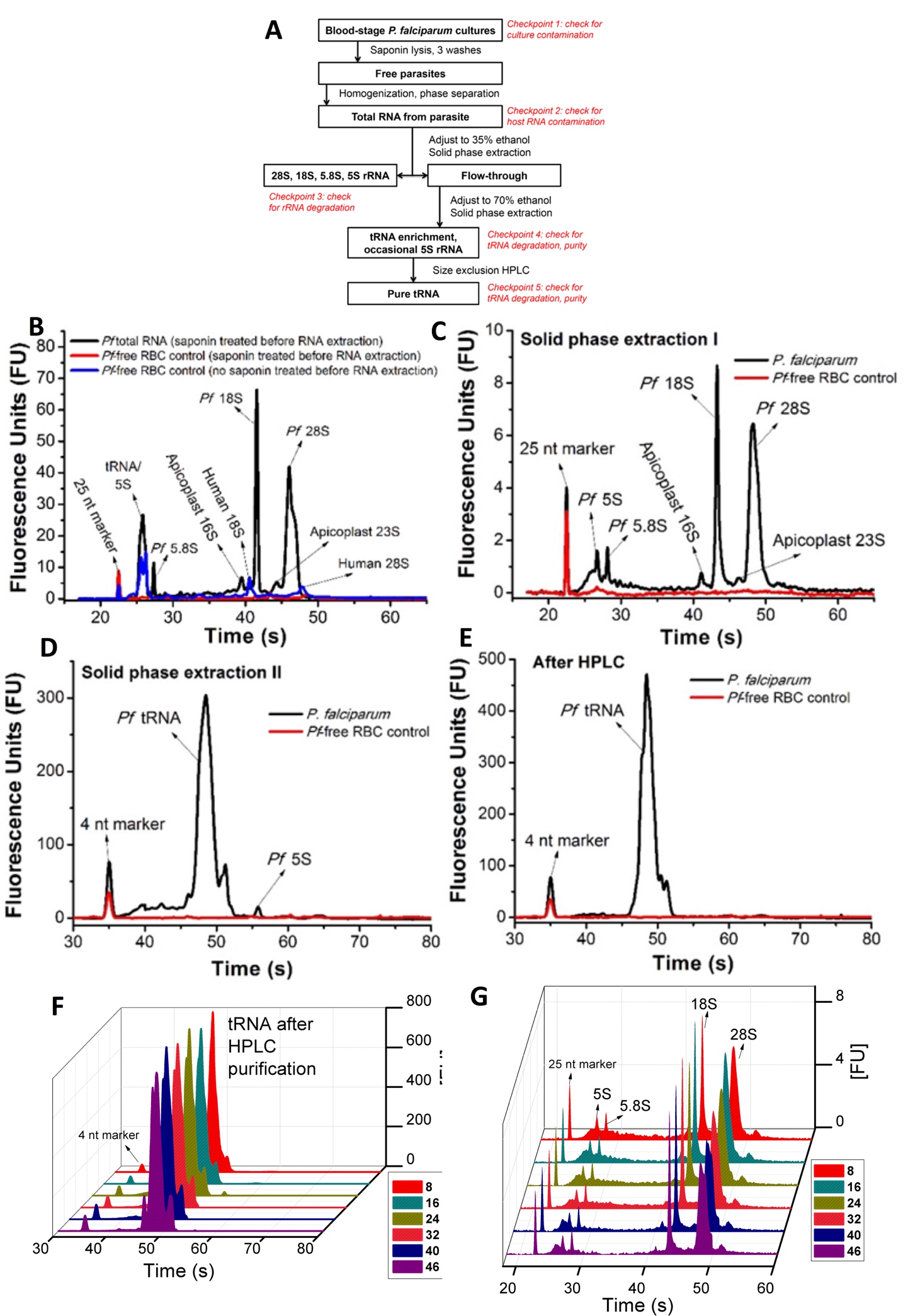


**Figure S1. Step-wise extraction, isolation, and quality examination of *P. falciparum* tRNA**. ***Related to Figures 1 and 2.*** (**A**) Experimental workflow to isolate pure tRNA from malaria parasites. Checkpoints for sample quality assessment are highlighted in red. The cultures were checked for contamination by mycoplasma and other microorganisms prior to starting an experiment, due to potential artifacts caused not only by microbial influences on parasite biology but also contributions of bacterial RNA, and associated modifications, to the parasite RNA. It is possible that the RBCs contain host RNA that may contaminate the parasite RNA pool. To exclude this possibility and minimize RNA derived from the host RBCs, we treated the cultures with saponin to selectively rupture RBC membranes (Gruenberg and Sherman, 1983) and release the parasites. Next, total RNA was extracted and the major non-coding RNA (ncRNA) species were assessed using a microfluidic-based Bioanalyzer, which rapidly provides both qualitative and quantitative information. (**B**) Total non-coding RNA species isolated from *P. falciparum* lysate (black) with comparison to parasite-free RBC lysate controls (saponin-treated, red; untreated, blue). (**C**) Elution of *P. falciparum* 28S, 18S, 5.8S and 5S rRNAs as well as putative apicoplast 23S and 16S rRNA retained in the first spin cartridge at 35% ethanol. Negative control using parasite-free RBC lysate is colored in red. (**D**) Elution of *P. falciparum* tRNA and minor amount of 5S rRNA retained in the second spin cartridge at 70% ethanol. Negative control using parasite-free RBC lysate is colored in red. (**E**) Bioanalyzer profile of tRNA isolate after HPLC purification. Parasite-free RBC control prepared by same procedures is colored in red. Identities and validation of each RNA species are based on sequence lengths on the Bioanalyzer RNA 6000 Pico Chips for panel **B**; Nano Chips for panel **C**; and Small RNA Chips for panels **D** and **E**. At least 3 independent experiments were repeated to confirm the reproducibility of the protocol. (**F, G**) Qualitative and quantitative consistency of (**F**) HPLC-purified tRNA and (**G**) the remaining non-coding RNA species (28S, 18S, 5.8S, 5S) in total RNA across the IDC, as measured on a Bioanalyzer.

**
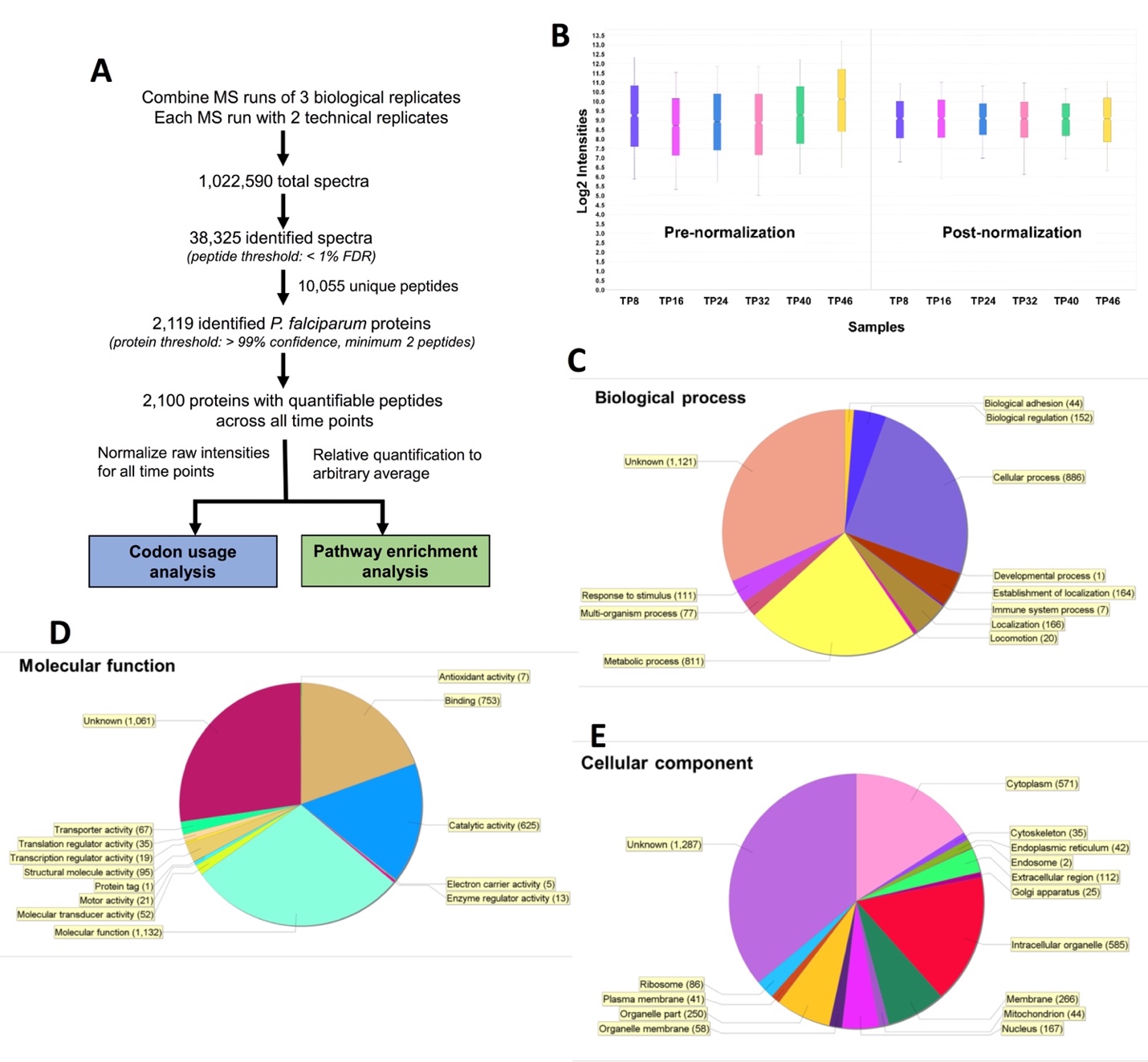
**

**Figure S2. Proteomic analysis of the *P. falciparum* IDC time-course.** ***Related to Figure 3***. (**A**) A total of 1,022,590 extracted spectra were matched to 38,325 identified spectra (peptide threshold: <1% FDR) which then mapped onto 2,119 *P. falciparum* proteins (protein threshold: >99% confidence, minimum 2 peptides) through database searching of *H. sapiens* and *P. falciparum* protein sequence databases (UniProtKB/Swiss-Prot). Among the identified *P. falciparum* proteins, 2,100 proteins were consistently quantified in every sample with no missing values of isotope-labeled peptides. The raw intensities of total quantified *P. falciparum* proteins at each sample were subjected to cross-sample normalization before quantitative comparisons across all channels. For time-course differential expression analysis of each protein, the respective intensities of the proteins in each channel were compared to the arbitrary average acquired across all samples. We selected the top 5% up-regulated proteins (n=100) at each time point for pathway enrichment analysis and codon usage analysis. (**B**) Pre- and post-normalization of the raw intensities for all proteins in each quantitative sample. Box plots displayed the values of log_2_ raw intensities of total proteins for corresponding sample with median and interquartile range. The cross-sample normalization was performed by Scaffold Q+ software. The X-axis of the raw figure is relabeled to indicate the respective time point of each sample. (**C-E**) Gene ontology analysis of the 2,100 identified *P. falciparum* proteins. The classification of the protein set was performed according to the first-level gene ontology terms: (**C**) Biological process, (**D**) Molecular function, and (**E**) Cellular component. The pie charts show the breakdown according to second-level classifications.


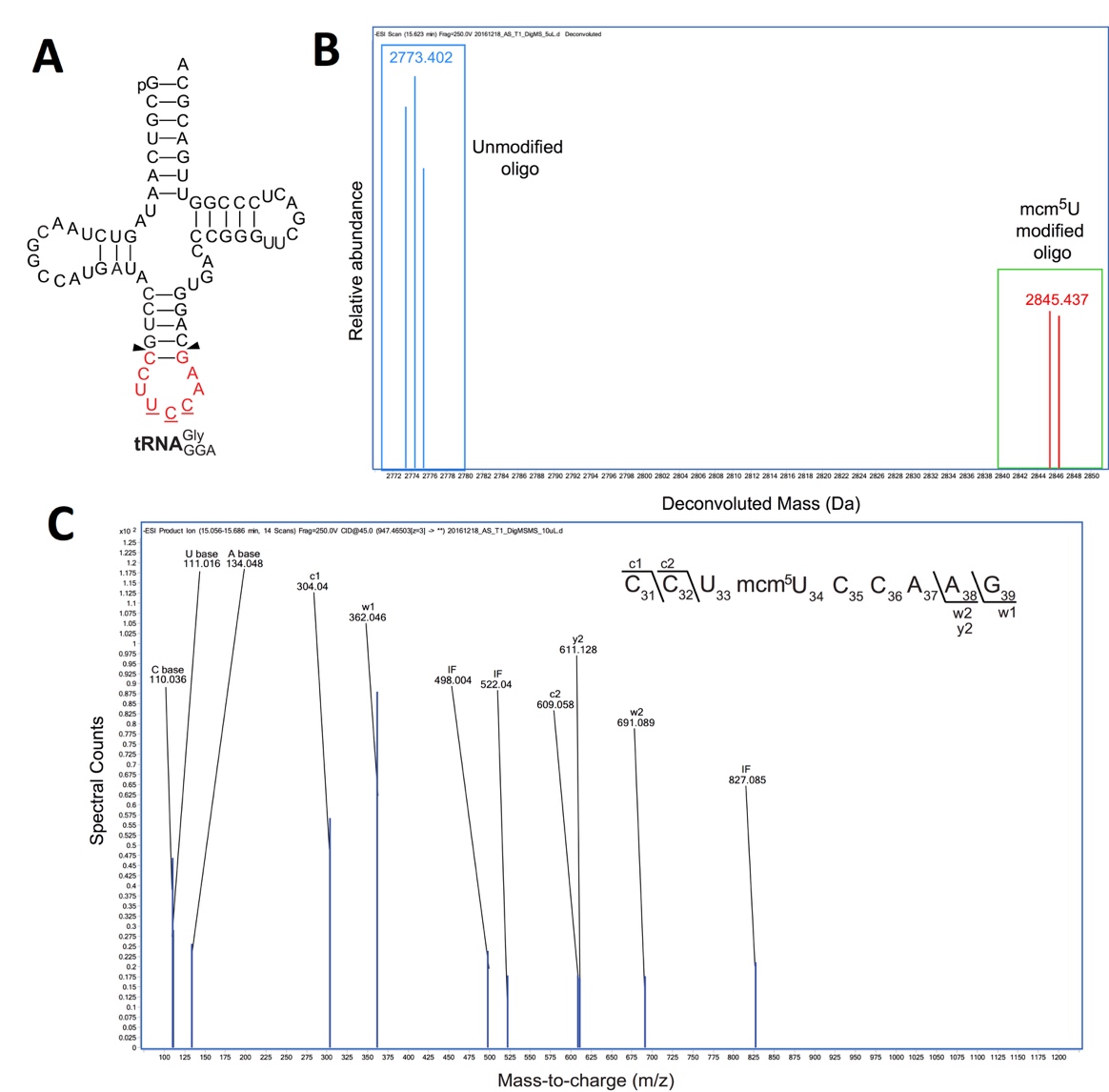


**Figure S3. *Plasmodium falciparum* Gly-tRNA^GGA^ contains wobble mcm^5^U. *Related to Figure 5*.** (**A**) Sequence of *Plasmodium falciparum* Gly-tRNA^GGA^. The arrows indicate T1 digestion sites. (**B**) LC-MS/MS was performed on T1 digested tRNA which upon deconvolution indicates precise mass of the unmodified (2773.402) and modified (2845.437) fragments. (**C**) Collision-induced dissociation of *m/z*1046.477^-3^ produces expected –c, –w and –y ions for the sequence CCU[mcm^5^U]CCAAG.

**Figure S4. *Related to Figure 5*.** Relative abundance of glutamic acid (GAA), glycine (GGA), and glutamine (CAA) isoacceptors as quantified by LCMS across ring, trophozoite and schizont stage of the parasite’s asexual lifecycle.

**Table S1. Modified ribonucleosides identified by LC-MS/MS analysis of *P. falciparum* tRNA hydrolysates**. ***Related to Figure 1***. Identification footnotes: **A**, MS^2^ fragments match reported data (Chan *et al.* 2010); **B**, validated by MS^2^ fragmentation of m^5^Cm standard eluting at 7 min, so annotated as m^4^Cm; **C**, validated by MS^2^ fragmentation of m^7^G and m^2^G standards eluting at 5 and 16.8 min, respectively, so annotated as m^1^G; **D**, validated by MS^2^ fragmentation of m^5^U and Um standards; **E**, MS^2^ fragments match reported data (Chan *et al.* 2010); **F**: Validated by MS^2^ and MS^3^ fragmentations of t^6^A and ms^2^i^6^A standards.

| Modified  ribonucleosides | Short name | Reten  time (min) | Theoretical [M+H]+ (m/z) | Observed molecular ion (m/z) | Major fragment ion (m/z) | Neutral loss (amu) | Quantifier SRM transition (precursor →  product) | Identification |
| --- | --- | --- | --- | --- | --- | --- | --- | --- |
| Dihydrouridine | D | 1.5 | 247.093 | 247.090 ± 0.005 | 115.048 ± 0.002 | 132.041 | 247.1 → 115.1 | Standard |
| Pseudouridine | Ψ | 1.6 | 245.078 | 245.077 ± 0.003 | 209.052 ± 0.004 | 36.034 | 245.1 → 209.1;  245.1 → 125.1 | Standard |
| 3-Methylcytidine | m^3^C | 2.5 | 258.109 | 258.106 ± 0.004 | 126.064 ± 0.002 | 132.042 | 258.1 → 126.1 | Standard |
| 5-Carbamoylmethyluridine | ncm^5^U | 2.6 | 302.099 | 302.093 ± 0.007 | 170.051 ± 0.002 | 132.042 | 302.1 → 170.0 | *A* |
| 5-Methylcytidine | m^5^C | 3.1 | 258.109 | 258.108 ± 0.004 | 126.065 ± 0.002 | 132.042 | 258.1 → 126.1 | Standard |
| 1-Methyladenosine | m^1^A | 3.2 | 282.120 | 282.117 ± 0.007 | 150.075 ± 0.003 | 132.042 | 282.1 → 150.1 | Standard |
| 7-Methylguanosine | m^7^G | 5.2 | 298.115 | 298.113 ± 0.005 | 166.071 ± 0.002 | 132.042 | 298.1 → 166.1 | Standard |
| 2'-*O*-Methylcytidine | Cm | 5.3 | 258.109 | 258.106 ± 0.004 | 112.050 ± 0.002 | 146.056 | 258.1 → 112.1 | Standard |
| Inosine | I | 6.6 | 269.089 | 269.088 ± 0.007 | 137.047 ± 0.002 | 132.042 | 268.1 → 137.0 | Standard |
| 5-Methyluridine | m^5^U | 6.7 | 259.093 | 259.088 ± 0.005 | 127.048 ± 0.002 | 132.042 | 259.1 → 127.0 | Standard |
| 2-Thiouridine | s^2^U | 7.4 | 261.055 | 261.051 ± 0.006 | 129.009 ± 0.008 | 132.042 | 261.0 → 129.0 | Standard |
| 2′-*O*-Methyluridine | Um | 9.6 | 259.093 | 259.092 ± 0.004 | 113.036 ± 0.002 | 146.056 | 259.1 → 113.1 | Standard |
| 3-Methyluridine | m^3^U | 10.1 | 259.093 | 259.089 ± 0.011 | 127.045 ± 0.005 | 132.044 | 259.1 → 127.0 | Standard |
| 5,2′-*O*-Dimethylcytidine | m^4^Cm | 11.1 | 272.125 | 272.122 ± 0.009 | 126.065 ± 0.002 | 146.057 | 272.1 → 126.1 | *B* |
| 2′-*O*-Methyladenosine | Am | 12.3 | 282.120 | 282.114 ± 0.003 | 136.059 ± 0.003 | 146.055 | 282.1 → 136.1 | Standard |
| 1-Methylguanosine | m^1^G | 14.5 | 298.115 | 298.111 ± 0.006 | 166.070 ± 0.002 | 132.041 | 298.1 → 166.1 | *C* |
| 5-Methoxycarbonylmethyl  uridine | mcm^5^U | 14.7 | 317.099 | 317.098 ± 0.010 | 185.054 ± 0.003 | 132.044 | 317.1 → 185.1 | Standard |
| 2'-*O*-Methylguanosine | Gm | 15.2 | 298.115 | 298.110 ± 0.007 | 152.056 ± 0.003 | 146.054 | 298.1 → 152.1 | Standard |
| *N*^6^-Methyladenosine | m^6^A | 16.5 | 282.120 | 282.115 ± 0.004 | 150.075 ± 0.002 | 132.04 | 282.1 → 150.1 | Standard |
| *N*^4^-Acetylcytidine | ac^4^C | 17.1 | 286.104 | 286.101 ± 0.005 | 154.056 ± 0.005 | 132.044 | 286.1 → 154.1 | Standard |
| *N*^2^-Methylguanosine | m^2^G | 17.7 | 298.115 | 298.112 ± 0.004 | 166.071 ± 0.005 | 132.041 | 298.1 → 166.1 | Standard |
| 5,2′-*O*-Dimethyluridine | m^5^Um | 18.8 | 273.109 | 273.108 ± 0.007 | 127.047 ± 0.005 | 146.061 | 273.1 → 127.1 | *D* |
| N2,N2-Dimethylguanosine | m^2,2^G | 19.7 | 312.131 | 312.127 ± 0.005 | 180.085 ± 0.003 | 132.041 | 312.1 → 180.1 | Standard |
| 5-Methoxycarbonylmethyl  -2-thiouridine | mcm^5^s^2^U | 20.3 | 333.076 | 333.075 ± 0.008 | 201.031± 0.005 | 132.044 | 333.1 → 201.0 | *E* |
| *N*^6^,*N*^6^-Dimethyladenosine | m^6,6^A | 20.5 | 296.136 | 296.131 ± 0.007 | 164.088 ± 0.008 | 132.043 | 296.1 → 164.1 | Standard |
| *N*^6^-Threonyl-carbamoyladenosine | t^6^A | 21.9 | 413.142 | 413.136 ± 0.005 | 136.062 ± 0.004 | 132.043 | 413.1 → 136.1;  413.1 → 281.1 | Standard |
| 2-Methylthio-*N*^6^-threonyl carbamoyladenosine | ms^2^t^6^A | 22.2 | 459.120 | 459.127 ± 0.020 | 327.084 ±0.010 | 132.043 | 459.1 → 327.1 | *F* |
| *N*^6^-Isopentenyladenosine | i^6^A | 22.5 | 336.167 | 336.159 ± 0.005 | 204.119 ± 0.003 | 132.04 | 336.2 → 204.1 | Standard |

**Table S2. Genome average usage for codons enriched in up- and down-regulated at 46 hr of the IDC**. ***Related to Figure 4.***

| ***Enriched in***  ***Up-reg’d Genes*** | | ***Enriched in***  ***Down-reg’d Genes*** | |
| --- | --- | --- | --- |
| ***Codon*** | ***Average***  ***Usage*** | ***Codon*** | ***Average***  ***Usage*** |
| Pro^CCA^ | 0.446 | Pro^CCT^ | 0.405 |
| Cys^TGC^ | 0.126 | Cys^TGT^ | 0.874 |
| Tyr^TAC^ | 0.114 | Tyr^TAT^ | 0.886 |
| Asn^AAC^ | 0.154 | Asn^AAT^ | 0.846 |
| Val^GTT^ | 0.396 | Val^GTG^ | 0.114 |
| Gly^GGA^ | 0.456 | Gly^GGG^ | 0.101 |

**Table S3. Enriched synonymous codons and their under-represented counterparts in late-stage (TP 40 and 46) up-regulated proteins. *Related to Figure 2 and 4.*** The listed codons on the left panel are more frequently used in late trophozoite/schizont (TP 40 and 46) up-regulated proteins in relative to down-regulated proteins. 9 out of 13 of the over-used codons are decoded by wobble-modified cognate tRNA iso-acceptors (shaded in aqua). Their anti-correlated (under-represented) synonymous codons are listed in the right panel. These under-represented codons are decoded by cognate tRNA species that are not known to be modified at wobble position, with tRNA^Ala(ncm5UGC)^ and tRNA^Ile(ΨAΨ)^ as exceptions. In addition, the tRNA species for decoding synonymous codons of Asn, His, Tyr, and Cys are not known to be wobbly modified (shaded in grey). Wobble position of codons (third base) and anticodons (34^th^ base) are underlined.

|  | **Up-regulated proteins at TP 40, 46** | | | **Down-regulated proteins at TP 40, 46** | | |
| --- | --- | --- | --- | --- | --- | --- |
| Amino  acid | Synonymous  codon | Cognate tRNA | Interaction | Synonymous  codon | Cognate tRNA | Interaction |
| Arg | AGA | tRNA^Arg(mcm5UGU)^ | Modified anticodon  A3:mcm^5^U34 | AGG | tRNA^Arg(CCU)^ | Watson-Crick  G3:C34 |
| Gly | GGA | tRNA^Gly(mcm5UCC)^ | Modified anticodon  A3:mcm^5^U34 | GGT | tRNA^Gly(GCC)^ | Wobble codon  U3:G34 |
| Glu | GAA | tRNA^Glu(mcm5s2UUC)^ | Modified anticodon  A3:mcm^5^s^2^U34 | GAG | tRNA^Glu(CUC)^ | Watson-Crick  G3:C34 |
| Pro | CCA | tRNA^Pro(ncm5UGG)^ | Modified anticodon  A3:ncm^5^U34 | CCT | tRNA^Pro(AGG)^ | Watson-Crick  U3:A34 |
| Ser | TCA | tRNA^Ser(ncm5UGA)^ | Modified anticodon  A3:ncm^5^U34 | AGT | tRNA^Ser(GCU)^ | Wobble codon  U3:G34 |
| Val | GTT | tRNA^Val(IAC)^ | Modified anticodon  U3:I34 | GTG | tRNA^Val(CAC)^ | Watson-Crick  G3:C34 |
| Leu | TTA | tRNA^Leu(ncm5UmAA)^ | Modified anticodon  A3:ncm^5^Um34 | CTA | tRNA^Leu(UAG)^ | Wobble anticodon  A3:U34 |
| Ala | GCT | tRNA^Ala(IGC)^ | Modified anticodon  U3:I34 | GCA | tRNA^Ala(ncm5UGC)^ | Modified anticodon  A3: ncm^5^U34 |
| Ile | ATC  ATT | tRNA^Ile(IAU)^ | Modified anticodon  C3:I34  U3:I34 | ATA | tRNA^Ile(ΨAΨ)^ | Modified anticodon  A3: Ψ34 |
| Asn | AAC | tRNA^Asn(GUU)^ | Watson-Crick  C3:G34 | AAT | tRNA^Asn(GUU)^ | Wobble codon  U3:G34 |
| His | CAC | tRNA^His(GUG)^ | Watson-Crick  C3:G34 | CAT | tRNA^His(GUG)^ | Wobble codon  U3:G34 |
| Tyr | TAC | tRNA^Tyr(GΨA)^ | Watson-Crick  C3:G34 | TAT | tRNA^Tyr(GΨA)^ | Wobble codon  U3:G34 |
| Cys | TGC | tRNA^Cys(GCA)^ | Watson-Crick  C3:G34 | TGT | tRNA^Cys(GCA)^ | Wobble codon  U3:G34 |

**Table S4.** **Electrospray series for unmodified and modified tRNA species presenting summary data for the T1-digested *Plasmodium falciparum* tRNA**. ***Related to Figure 5.***

| **tRNA**  **species** | **Sequence of RNAseT1 fragment** | **Calc’d Mass (Mono-isotopic)** | **Obs’d Mass (Decon-voluted)** | **Diff’nce (Δppm)** | **HPLC Ret’n time (min)** | **Negative Ions** | | **Charge State** |
| --- | --- | --- | --- | --- | --- | --- | --- | --- |
|  |  |  |  |  |  | **Calc’d** | **Obs’d** |  |
| Gln-CAA | ACUUUG | 1835.259 | 1835.264 | 2.724 | 14.343 | 610.745 | 610.751 | -3 |
|  |  |  |  |  |  | 916.621 | 916.635 | -2 |
| Modified  Gln-CAA | ACU**mcm^5^s^2^U**UG | 1923.257 | 1923.262 | 2.599 | 13.64 | 640.078 | 640.075 | -3 |
|  |  |  |  |  |  | 960.621 | 960.623 | -2 |
| Glu-GAA | CUUUCACCCG | 3055.423 | 3055.425 | 0.654 | 15.726 | 762.848 | 762.951 | -4 |
|  |  |  |  |  |  | 1017.466 | 1017.474 | -3 |
| Modified  Glu-GAA | CU**mcm^5^s^2^U**UCACCCG | 3143.421 | 3143.429 | 2.545 | 16.248 | 784.847 | 784.851 | -4 |
|  |  |  |  |  |  | 1046.799 | 1046.801 | -3 |
| Gly-GGA | CCUUCCAAG | 2773.409 | 2773.402 | -2.5239 | 15.623 | 923.462 | 923.462 | -3 |
|  |  |  |  |  |  | 692.344 | 692.346 | -4 |
| Modified  Gly-GGA | CCU**mcm^5^U**CCAAG | 2845.43 | 2845.437 | 2.46 | 15.623 | 947.469 | 947.141 | -3 |
|  |  |  |  |  |  | 710.349 | - | -4 |

**Table S5. Codon usage and cytoplasmic tRNAs in *P. falciparum*. *Related to Fig. 4.*** Anticodon loop sequences (32-38) of the 43 tRNA species in *P. falciparum* (1 initiator, 42 elongator). Anticodons in red, wobble 34 underlined. Presumed wobble modifications underlined; based on *S. cerevisiae* ([Agris et al., 2007](file:///F:\Manuscripts%202015\tRNA%20Modifications%20in%20Pfalciparum\Supplemental%20Information\Table%20S2\Table%20S2.docx#_ENREF_4); [Grosjean et al., 2010](file:///F:\Manuscripts%202015\tRNA%20Modifications%20in%20Pfalciparum\Supplemental%20Information\Table%20S2\Table%20S2.docx#_ENREF_81); [Jackman and Alfonzo, 2013](file:///F:\Manuscripts%202015\tRNA%20Modifications%20in%20Pfalciparum\Supplemental%20Information\Table%20S2\Table%20S2.docx#_ENREF_103); [Roth, 2012](file:///F:\Manuscripts%202015\tRNA%20Modifications%20in%20Pfalciparum\Supplemental%20Information\Table%20S2\Table%20S2.docx#_ENREF_186)). *NA: not applicable. NR: no report.*

| **Amino**  **acid** | **Synonymous codons**  **(mRNA: 5'→3')** | **Cognate tRNA iso-acceptor (32 to 38)** | **Genome average usage** | **Presumptive wobble modification** |
| --- | --- | --- | --- | --- |
| Ala | GCA | TTTGCAT | 0.425 | ncm^5^UGC |
|  | GCC | NA | 0.107 | NA |
|  | GCG | TTCGCAT | 0.052 | NR |
|  | GCU | TTAGCAT | 0.416 | IGC |
| Arg | AGA | CTTCTAA | 0.614 | mcm^5^UCU |
|  | AGG | TTCCTAA | 0.158 | NR |
|  | CGA | CTTCGGA | 0.087 | NR |
|  | CGC | NA | 0.018 | NA |
|  | CGG | NA | 0.011 | NA |
|  | CGU | CTACGGA | 0.113 | ICG |
| Asn | AAC | CTGTTAA | 0.154 | NR |
|  | AAU | NA | 0.846 | NA |
| Asp | GAC | CTGTCAC | 0.143 | NR |
|  | GAU | NA | 0.857 | NA |
| Cys | UGC | CTGCAGA | 0.126 | NR |
|  | UGU | NA | 0.874 | NA |
| Gln | CAA | CTTTGAA | 0.860 | mcm^5^s^2^UUG |
|  | CAG | CTCTGAC | 0.140 | NR |
| Glu | GAA | CTTTCAC | 0.862 | mcm^5^s^2^UUC |
|  | GAG | CTCTCAC | 0.138 | NR |
| Gly | GGA | CTTCCAA | 0.456 | mcm^5^UCC |
|  | GGC | TTGCCAT | 0.043 | NR |
|  | GGG | NA | 0.101 | NA |
|  | GGU | NA | 0.400 | NA |
| His | CAC | CTGTGGA | 0.154 | NR |
|  | CAU | NA | 0.846 | NA |
| Ile | AUA | CTTATGT | 0.545 | ΨAΨ |
|  | AUC | NA | 0.066 | NA |
|  | AUU | CTAATAA | 0.389 | IAU |
| Leu | CUA | TTTAGGC | 0.076 | NR |
|  | CUC | NA | 0.023 | NA |
|  | CUG | GTCAGAT | 0.020 | NR |
|  | CUU | TTAAGGC | 0.121 | NR |
|  | UUA | CTTAAGA | 0.622 | ncm^5^UmUA |
|  | UUG | CTCAAGA | 0.138 | m^5^CAA |
| Lys | AAA | CTTTTAA | 0.822 | mcm^5^s^2^UUU |
|  | AAG | CTCTTAA | 0.178 | NR |
| Met | AUG | CTCATAA | 1.000 | NR |
| Phe | UUC | CTGAAGA | 0.162 | GmAA |
|  | UUU | NA | 0.838 | NA |
| Pro | CCA | TTTGGGT | 0.446 | ncm^5^UGG |
|  | CCC | NA | 0.104 | NA |
|  | CCG | TTCGGGT | 0.046 | NR |
|  | CCU | TTAGGGT | 0.405 | NR |
| Ser | AGC | CTGCTAA | 0.059 | NR |
|  | AGU | NA | 0.294 | NA |
|  | UCA | CTTGAAA | 0.277 | ncm^5^UGA |
|  | UCC | NA | 0.085 | NA |
|  | UCG | CTCGAAA | 0.047 | NR |
|  | UCU | CTAGAAA | 0.238 | IGA |
| Thr | ACA | CTTGTAA | 0.529 | ncm^5^UCU |
|  | ACC | NA | 0.116 | NA |
|  | ACG | TTCGTAA | 0.092 | NR |
|  | ACU | CTAGTAA | 0.263 | IGU |
| Trp | UGG | CTCCAGA | 1.000 | CmCA |
| Tyr | UAC | CTGTAGT | 0.114 | NR |
|  | UAU | NA | 0.886 | NA |
| Val | GUA | CTTACAC | 0.425 | ncm^5^UAC |
|  | GUC | NA | 0.065 | NA |
|  | GUG | CTCACAC | 0.114 | NR |
|  | GUU | CTAACAC | 0.396 | IAC |
